# Targeting Complex Orthography in the Treatment of a Bilingual Aphasia with Acquired Dysgraphia: The Case of a Malay/English Speaker with Conduction Aphasia

**DOI:** 10.3390/bs10070109

**Published:** 2020-07-05

**Authors:** Mohd Azmarul A Aziz, Rogayah A Razak, Maria Garraffa

**Affiliations:** 1 Speech Therapy Unit, Cheras Rehabilitation Hospital, Kuala Lumpur 56000, Malaysia; mohdazmarul@moh.gov.my; 2 Clinical Research Centre, Ministry of Health Malaysia, Kuala Lumpur 56000, Malaysia; 3 Centre for Rehabilitation & Special Needs Studies, Faculty of Health Science, Universiti Kebangsaan Malaysia, Kuala Lumpur 50300, Malaysia; 4 Department of Psychology, School of Social Sciences, Heriot-Watt University, Edinburgh Eh14 4AS, UK

**Keywords:** bilingual aphasia, dysgraphia, anagram and copy treatment (ACT), multiple-baseline design, aphasia

## Abstract

Background: Disruption of spoken language in people with aphasia tends to interfere with the ability to write, which is referred to as dysgraphia. This study examined the effectiveness of the anagram and copy treatment (ACT), administered in English on a bilingual Malay/English patient with conduction aphasia (GM). ACT is the arrangement of component letters presented in scrambled order (i.e., an anagram) so that the patient could use the letters to form target words, followed by repeated copying of the word. Methods: A single-subject multiple-baseline design was used with sets of English words (both nouns and verbs) sequentially targeted for treatment. Prior to the treatment, a series of single word writing and reading baselines were conducted in two languages: English and Malay. The ACT treatment was done in English, the language reported as more dominant for reading by the patient. Probes assessing generalizations to untrained pictures were presented at 8th, 13th, and 18th sessions. Results: GM showed steady and incremental improvement in the writing of trained nouns and verbs, with generalizations to untrained English nouns and verbs. Conclusions: Single word writing treatment in a non-transparent language may improve dysgraphia among adults with bilingual aphasia through the administration of a structured and systematic treatment.

## 1. Introduction

Aphasia is a multi-modal language disorder consisting of a combination of speech and language disorders caused by damage to the brain [1]. Approximately 21–38% of acute stroke patients suffer from some form of aphasia [2]. Among the different types of aphasia, conduction aphasia is a fairly rare type of aphasia that is caused mainly due to lesions in the supramarginal gyrus along with damage to the white matter that comprises the arcuate fasciculus. Usually, conduction aphasia is caused by an embolic stroke in the parietal or posterior region of the temporal lobe [3]. Conduction aphasia is characterized by fluent verbal output, almost normal levels of auditory comprehension, and significant impairments in repetition [4]. Secondary characteristics also appear based on the severity level and these characteristics include anomia, impairments in reading, and variable levels of writing difficulties (dysgraphia). In this short report, we will focus on the writing disorder often reported in aphasia and its treatment.

Speech and language therapies available to treat dysgraphia are often based on impairment level or on providing compensatory strategies to patients [5]. Impairment-based therapies for dysgraphia focus on lexical aspects, or training of single words through repetitive spelling and dictation, for example, in copying tasks [6]. Some impairment therapies also focus on retraining phoneme-grapheme conversational skills in patients who suffer from dysgraphia due to phonological deficits [7]. When one of the modalities is more severely affected than the other, therapy is sometimes shifted towards a compensatory approach whereby the therapy is focused on the modality that is more amenable to treatment. Treatment focuses on residual abilities and maximizing communication by improving performances of less severely affected abilities [8]. For the cases of impairment to both modalities, speaking and writing therapies can be combined for effective treatment of aphasia [9]. For conduction aphasia, the speaking and writing difficulties are commonly not as severe as some other forms of aphasia, therefore, can be treated more easily compared to speech with therapy [10].

Previous studies on treatment of writing and speaking difficulties for patients with acquired aphasia have shown the effectiveness of treatment protocols targeted for written communication as well as spoken language. However, there is a scarcity of literature that focuses on a treatment that combines both written and spoken modality, with most of the studies using a protocol based on compensatory-based strategies. A combination of therapy is important especially when there is potential that both aspects of communication can be enhanced [9], especially in cases like that of conduction aphasia. Therefore, the present study is aimed at examining the effectiveness of a treatment technique called anagram copy treatment (ACT) which focuses on improving single word vocabulary for both written and verbal aspects of communication on a single patient with acquired conduction bilingual aphasia. This study will feature both modalities involved in ACT. The ACT protocol was administered in English, a language with opaque grapheme/phoneme conversion. At the same time, the specificity of language: generalizations on the Malay language, the first language of the patient, will also be touched on briefly. Before presenting the data on the ACT therapy of the single case, we will introduce the literature on ACT and some aspects discussed in bilingual dysgraphia. We will conclude the introduction with a short snapshot on the Malay orthography. This section will be functional to a comprehensive characterization of the language of the patient, although the treatment proposed in the study will be in English.

### 1.1. Treatment Studies on Acquired Dysgraphia

Studies have shown successful treatment of acquired dysgraphia using the anagram and copy treatment therapy (ACT) [11]. This therapy addresses impairments on both lexical and non-lexical spelling routes. ACT involves presentation of anagram letters of a target word so that the patient will be asked to rearrange the letters to form targeted words. After accurate rearrangement, the procedure involves writing down the word so that the correct spelling can be reinforced. Studies adopting ACT demonstrated the effectiveness of ACT on treating dysgraphia in patients with aphasia [5]. Similar results were obtained when ACT and CART (copy and recall treatment) were combined and tested on single word spelling and functional writing [12]. Consistent to this, a study that combined both ACT and CART showed that three months of therapy improved the writing skills of three individuals with severe aphasia who exhibited dysgraphia [13]. Another study adopting a method similar to ACT used a “copy and recall and note-taking practice” method that targeted writing to dictation of single words and note-writing ability. The study found significant improvements in writing to dictation of trained and untrained words and note taking ability [14]. The cognitive processing model of language processing is the framework used to better understand how the above therapies produced gains in writing and spelling [12]. This model will be the underlying conceptual framework of the current study.

**Figure 1:**
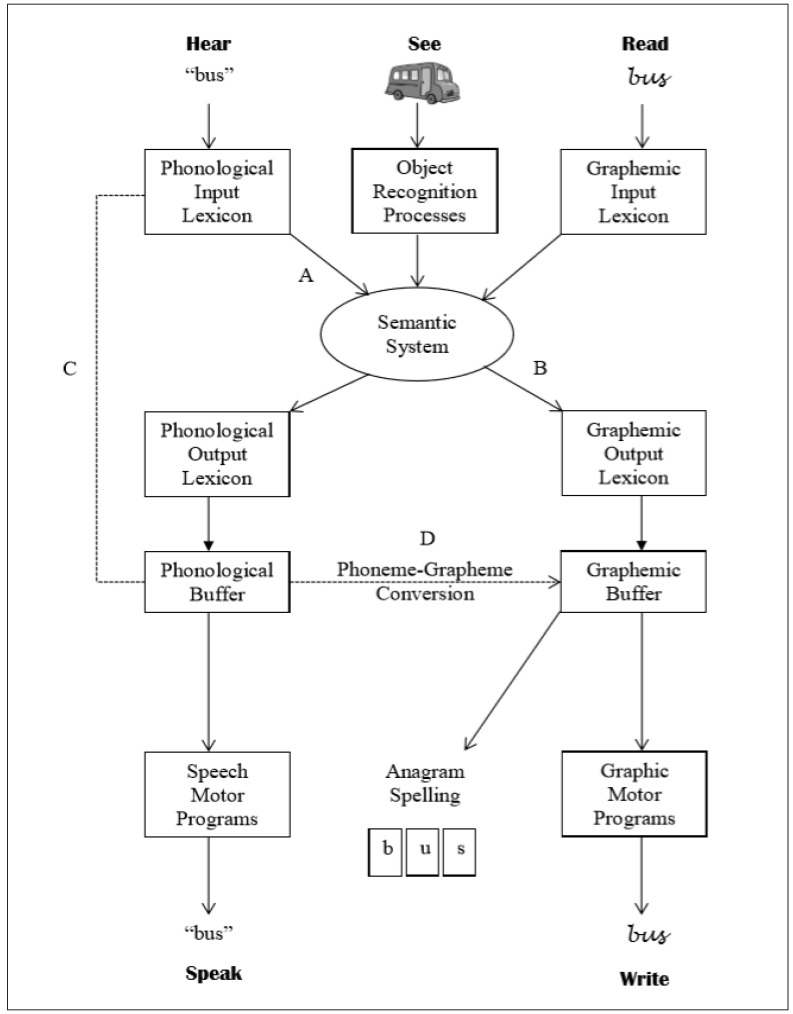
Cognitive processing model of language processing.

According to this framework, memory store of spellings of learnt words are known as “graphemic output lexicon”. For many languages, such as Malay, this can be accessed directly from the semantic system, which is connected to the phonological input lexicon, object recognition process, and graphemic input lexicon, as shown in Figure 1. Pathway B, which is the link between the semantic system and graphemic output lexicon, plays a significant role in conceptual writing activities such as spontaneous writing and naming. Writing to dictation is also supported by the lexical-semantic spelling route, by connections between “phonological input” and “graphemic output lexicon” via the semantic system (Path A and B). This graphemic representation must be strong enough to retain in the graphemic buffer, as the correct shapes of letters are chosen for writing or the component letters are organized for anagram spelling. When a word is written, the abstract graphemic information is changed to letter shapes or allographs, which are created by movements made during handwriting that are guided by graphic motor programs as seen in Figure 1.

The main idea of this study is to work on the language that is making systematic use of the phoneme-grapheme conversation route (English) and to monitor changes in writing abilities in both languages.

Spelling can also be accomplished in a way that does not require semantic representation, and is only dependent on sound-to-letter or phoneme-to-grapheme conversions. This procedure involves division of phonological input into its component phonemes, and translating each phoneme to its corresponding grapheme by using pathway C and D. For many aphasic patients, the locus of the impairment is the connection between pathways; therapies such as CART, ACT, and other speech and language therapies focused on (re)establishing the connections between such pathways [12].

### 1.2. Bilingual Dysgraphia

Few studies have been conducted on bilingual dysgraphia, with the majority of them reporting evidence for a language independent disorder with similar pattern of errors across languages [15,16]. Patterns of errors depending on classical psycholinguistics dimensions (word frequency/imageability/grammatical class) were reported for many languages including Arabic, Dutch, Finnish, French, Italian, Greek, Japanese, Korean, and Spanish, with no differences across orthographic systems (more or less transparent). A recent study on dysgraphia in a bilingual Greek/English aphasic speaker [17] reported a similar pattern of errors in both languages, with poor spelling of non-words compared to words in English as well as Greek. These patterns of non-word spelling disorder support the hypothesis that in bilingual dysgraphia both languages are impaired, at least in studies on non-lexical spelling. This point is confirmed further by a study on an English/Cantonese biscriptal patient [18], with similar patterns of errors in both scripts. An interesting issue in bilingual dysgraphia is how the disorder in writing is manifested according to the transparency of the language. This question has important clinical implications. For example, deep dysgraphia in English has been proposed to reflect a damage of the non-lexical spelling pathway with over-reliance on spelling of the lexical-semantic pathway. This difference could be the outcome of the opaque English script and as the prediction for no errors at lexical level for a more transparent language (such as Malay in this study). A clinical design for treatment of bilingual dysgraphia could capitalize on cross language differences and consequent difference in the source of the impairment. In languages such as English, dysgraphic patients misspell irregularly spelled words e.g., eyes → AIS and make errors with homophone-word mixing in writing as a consequence of the non-lexical spelling damage. These errors are more visible due to the opaque script. In the case of an acquired dysgraphia, is it possible that the otherwise hidden processes of a language, for example, the contribution of the non-lexical level for more transparent languages, are now essentials to overcome a non-expert system? For example, English does require both phonological and lexical knowledge in order to retrieve a word. This is not often the case for more transparent languages, such as Spanish or Malay as in this study. Some studies on both dysgraphia and dyslexia have actually reported the operation of lexical-semantic reading to be necessary, for example, for Turkish or Spanish patients [19,20]. Bilingual dysgraphia can be an opportunity to investigate the source of the impairment and gain a different angle of the impairment not provided by the spoken modality or by a dysgraphia in a monolingual speaker [9].

We will now introduce some basic aspects of the Malay orthography to gather a more complete picture of the patients’ competences.

### 1.3. Malay Orthography

The Malay language (henceforth Malay) belongs to the group of languages from the Malayo-Polynesian branch of the Austronesian family of languages [21]. Malay was the lingua franca in the South East Asia region in the 1500s, however, in current times Malay, a language spoken by 250 million people, is spoken in four countries in the region—Malaysia, Indonesia, Singapore, and Brunei. Malay has shallow alphabetic orthography, simple syllable structures, and transparent affixation—characteristics that contrast sharply with those of English [22]. Malay uses a Rumi or Romanized writing system based on the Latin script. The phonological structure of Malay words can be described in terms of both syllable and phonic structures. Words have simple syllable structures but with varying levels of phonic structure. For example, the word masa/time has both a simple syllable and a simple phonic structure (CV [ma] + CV [sa]), whereas the word sungai/river has a more complex phonic structure (CV [su] + CVV [ɳai]) with digraph and diphthong [22]. Shallow orthography means that there is an isomorphic relationship between spelling and sound i.e., where the mappings between orthography and phonology are transparent and predictable compared to languages with deep orthography such as English, where the mapping between orthography and phonology is more complex—same graphemes represent different sounds across different contexts [21].

### 1.4. Rationale for the Current Study

As seen from the literature reviewed above, previous studies have shown that impairment-based and compensation-based strategies have improved performances in writing and speaking modalities of patients with aphasia with different levels of dysgraphia. Therapies such as ACT have effectively resulted in the better retaining of single word vocabulary for patients with acquired aphasia. However, there seems to be limited literature concerning the combination of therapy which targets the improvement of both written and spoken aspects in bilingual aphasic patients who suffer from dysgraphia. More specifically, very limited studies have been conducted on patients with bilingual conduction aphasia. Additionally, despite the significance of written communication, especially in an age that involves the use of written communication via different languages, there is limited research into spelling therapies for individuals with acquired bilingual dysgraphia [23]. Therefore, the current study aims to fill this gap by focusing on examining the effectiveness of a writing treatment (ACT) in English on rebuilding and maintenance of single word vocabulary for both written and verbal aspects of communication of a stroke patient with conduction aphasia who exhibits dysgraphia in both languages.

## 2. Materials and Methods

**Figure 2:**
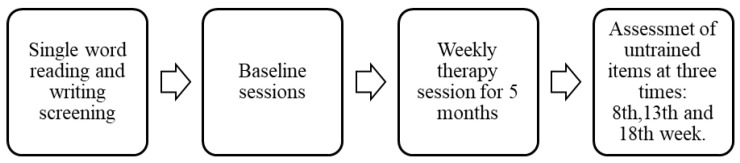
Timeline of the single subject multiple-baseline design.

A single-subject multiple-baseline design was employed to examine the effects of ACT. Treatments were administered by the examiner who is a qualified speech-language pathologist. The ACT procedure was implemented following standard procedures in [12]. The participant attended once-weekly therapy sessions over a period of five months. Two sets of 50 English words (nouns and verbs each) were selected after a ranking based on the frequency of use in the patient daily activities. Most of the words were functional to the participant’s daily activities from more familiar to less familiar, including common nouns, proper nouns, and common verbs. This method was preferred compared to the adoption of frequency from a standard database due to the fact that all databases available from word frequencies are calculating properties of use in monolingual speakers and do not take into account the difference in use of the language due to bilingualism. All selected words were single words from one to three syllables, and from three to ten letters. Then, a minimum of 30 words aka trained words (three sets of ten words) (see Appendix A) was targeted for treatment. The trained words were selected from the incorrect responses from the set of 50 words. These words were mainly for training purposes in order to help the participant to rebuild the process of writing and producing single words vocabulary. Figure 2 shows the timeline of the single subject multiple-baseline design used in this study.

In terms of the study design, two options were available for single-case experimental treatment studies [24]: the single-subject experimental design (SSED) and the single-case study design (SCSD). The current study has a few of the main features adopted in single-case treatments, for example:

(1) Pretreatment baseline sessions continue until the subject achieves “stable” performance.
(2) Performance on treated and untreated items is probed frequently, often at every session.
(3) Treatment continues until the subject achieves a criterion level (e.g., 80% on three successive probes).
(4) The effectiveness of therapy is compared statistically.
(5) Typically, the number of items in the treatment and control conditions is small (usually 6–20 items).

In the present study, we chose a slightly similar approach as described above. However, the selection of stimuli for treatment required two pretreatment phases [24]: first, a selection phase where difficult items are selected from a larger set and, second, a baseline phase where items are tested again on at least two (and preferably more) occasions to determine the pre-treatment level of performance to avoid from problems described in the literature as ‘regression to the mean’ [24]. It is important to keep in mind that unlike the study reported in the literature [11], where the participants were monolingual speakers, being a bilingual speaker with dysgraphia in two languages requires a different set of considerations and this is more evident in a protocol such as the ACT treatment, a complex task aiming at consolidating the representation of words functional to the patient. In order to avoid a potential confound in this pilot study, ACT was adopted to establish a more systematic baseline at each point. If the results of this pilot study adhere to what was reported in other studies adopting the ACT, a follow up study will adopt what has been suggested in the literature for monolinguals [13].

### 2.1. Anagram and Copy Treatment (ACT)

**Figure 3:**
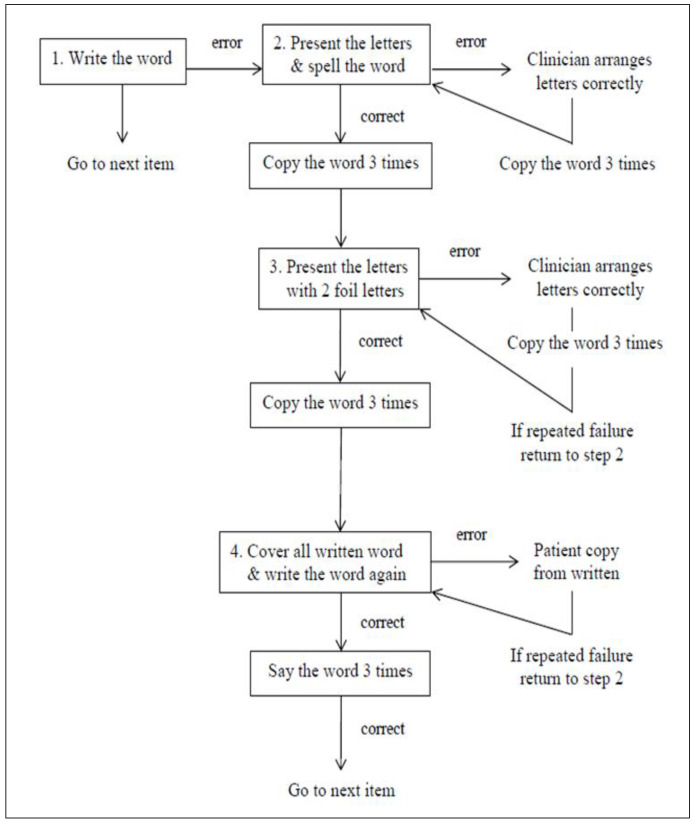
Schematic representation of the anagram and copy treatment (ACT) adapted from [12].

Based on [12] description, the ACT protocol is a cueing hierarchy used to elicit correct spelling of target words. As shown in Figure 2, the participant was asked to spell a word depicted by a representative picture. Semantic information was provided by the clinician as the picture is presented to maximize the likelihood that graphemic representations were connected to semantics. If the target word was not written correctly, component letters were presented in random order for the participant to manipulate in order to spell the word. Following successful arrangement of the letters, repeated copying of the word was required (three times), as a way to strengthen memory for spelling. Immediately following repeated copying of the word, all written examples of the target were covered or removed, and recall of spelling was tested successively three times. As noted in Figure 3, corrective feedback was provided by the clinician and repeated copying of the target word occurs throughout the protocol.

### 2.2. Participant

GM is a 62-year-old, right-handed man who experienced a left hemisphere stroke at the age of 60 that resulted in right hemiparesis and difficulty in speaking and writing. A CT head scan revealed a non-hemorrhagic infarct in the left middle cerebral artery (post thrombolysis). GM is a bilingual Malay and English speaker who taught as an academician for more than 25 years. After retiring from academia, he secured an offer to work as a contract professor at a private university but unfortunately had a stroke just before he started his new job. Prior to the stroke event, he was reported to have had difficulties in communication. His speech production was limited, effortful, halting, and mainly consisted of short phrases (mostly single words) with significant word-finding difficulty. Repetition skills were difficult with increased syllables in words and sentences, and the writing was also poor. However, his auditory comprehension at single words and sentences, and reading (both reading aloud and reading comprehension) were good (see next section for the formal assessments). GM’s language profile in both languages was recorded with the bilingual language profile, The Language Experience and Proficiency Questionnaire (LEAP-Q) [25]. The outcome of the LEAP-Q showed a dominance of Malay as the first language, a preference for English in reading and writing, and a balanced use of both languages in the spoken modalities. English was learnt as a young adult (age of acquisition was 10 years old), spoken at home, and with a high level of proficiency. The participant gave his informed consent for inclusion before he participated in the study. The study was conducted in accordance with the Declaration of Helsinki, and the protocol was approved by the Ethics Committee of Medical Research and Ethics Committee, Ministry of Health Malaysia (NMRR-20-810-54771).

### 2.3. Pre-Treatment Assessments

Prior to the initiation of writing treatment, pre-treatment assessments were conducted: (1) Western-Aphasia Battery-Revised (WAB-R) in both languages [26]—to determine the type and severity of aphasia as well as to identify which language was more preserved after stroke; (2) Pyramid and Palm Tree Test (PPTT) [27]—to assess semantic access from words and pictures and determine the degree to which the participant can access meaning from pictures and words; and (3) Psycholinguistics Assessment of Language Processing in Aphasia (PALPA) [28]—to assess and determine the ability of the participant in reading, writing, and naming skills. Writing to dictation was assessed using PALPA Subtest 31, 39, 40, 41, 42, 43, 44, 45, and 46, which controls for imageability, frequency, regularity, length, grammatical class, and morphological complexity effects of the stimulus items. PALPA Subtest 48 was used to assess single-word reading comprehension where the participant matched written word to a target picture of five selections. Recognition of written words was assessed using a visual lexical decision task (PALPA Subtest 25), which required matching identification of 60 real words presented along with 60 nonword distractors. PALPA Subtest 53 was used to name common pictures presented to the participant.

The results of WAB-R showed that GM was diagnosed with moderate conduction aphasia for English and moderate Broca’s aphasia for Malay, whilst the PPTT results showed good access to the semantic system. However, most of the PALPA tests selected showed results in poor spelling performance (below 60% correct), except for visual lexicon decision (PALPA 25) and written word picture naming (PALPA 48) with a score of more than 85% correct. Single word writing ability was extremely limited for writing to dictation and surprisingly an oral reading task (PALPA 31) also demonstrated poor performance (58%) despite intact reading aloud in the WAB-R test.

### 2.4. Data Collection

A set of 50 words, each for nouns and verbs were presented to determine the baseline performance for spoken and written words production and to select trained items for the treatment. The tasks were conducted in English. GM was able to name 25/50 (50%) and write 22/50 (44%) correct in the noun task, whilst he was able to name 21/50 (42%) and 19/50 (38%) correct in the verb task. This showed that GM’s nouns production was better than verbs production in spoken and written naming tasks, although both were equally poor in performance (less than 50%).

Following the treatment procedure suggested by [11], spelling of target words was probed at the beginning of each treatment session by showing the picture and asking the participant to write the appropriate word. These data were examined to determine baseline performance and to demonstrate their responses to treatment. Once a set of words was entered into treatment, the criterion for mastery of a word set was 80% correct (e.g., eight out of a set of ten words). Word sets were sequentially entered into treatment following achievement of criterion with the preceding set.

## 3. Results

### 3.1. Treatment Procedure and Results

**Figure 4:**
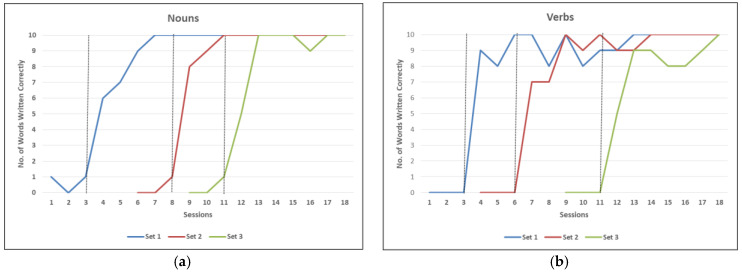
(**a**,**b**) Pre- and post-treatment on nouns (**a**) and verbs (**b**) over 18 sessions in English. The dashed lines show the baseline score for each set before the treatment begins.

**Figure 5:**
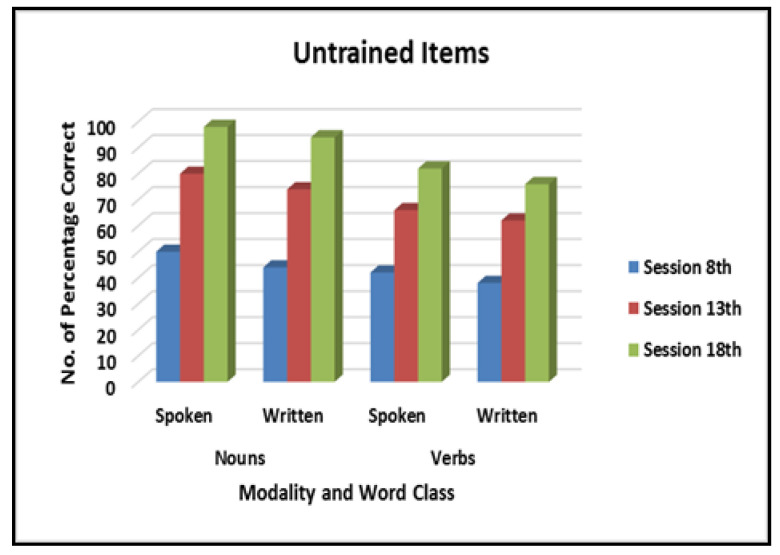
Performance of untrained nouns and verbs in spoken and written naming at each baseline in English.

From a total of 60 words (30 nouns, 30 verbs; the words ranged in length from one to three syllable words and from three to ten letters) selected for the writing treatment, words were divided into three sets of ten nouns and ten verbs. Prior to treatment, GM was unable to spell 50% of the words correctly. The ACT protocol was employed during the weekly treatment sessions. The treatment was initiated for the set one first for both nouns and verbs. His spelling for the first set of ten words was poor over four sessions, with almost no correct responses. At the beginning of session five, improvement was seen and by 7th session, the patient showed steady improvement reaching 100% correct in both word classes. After the 11th session of treatment, GM had reached criterion for correct written spelling of all three sets of words. As reported in Figure 4 and Figure 5, GM showed stable baseline performance for all word sets for both nouns and verbs prior to the initiation of treatment until the 18th session. Not only did he show rapid improvement in written spelling of target words (trained words) as they entered into treatment, he also showed improvement on untrained words, spoken (49/50, 98%) and written (47/50, 94%) for nouns and spoken (41/50, 82%) and written (38/50, 76%) for verbs. The treatment improvement was effective in all modalities, as reported in Figure 5, with an incremental effect across sessions.

### 3.2. Pre- and Post-Treatment Assessment Results

Following treatment, GM was reassessed after two weeks to determine whether there were any changes in the type and severity of aphasia while his writing ability was reassessed using the PALPA subtests. Results of WAB-R on both languages demonstrated that his severity of aphasia had improved from moderate to mild and the type of aphasia has evolved from conduction (in English) and Broca’s (in Malay) to anomic aphasia (see Table 1). His writing ability had shown marked improvement in all the PALPA subtests (see Table 2). It is interesting to note that both nouns and verbs in English had similar results, with no apparent grammatical category effect (see Figure 4a,b). Since nouns and verbs were selected according to the patient functionality and not based on psycholinguistics measures, we will not discuss in detail the lack of grammatical category effect.

Further statistical analysis using a Wilcoxon signed-rank test was done to determine the significance of improvement for the WAB-R, PALPA, and PPTT pre- and post-treatment. The results showed that there was a significant improvement for the WAB-R in both English (*Z* = −2.668, *p* < 0.05) and Malay (*Z* = −2.805, *p* < 0.05). However, there was no significant difference in the magnitude of improvement between English and Malay WAB-R (*Z* = −1.590, *p* = 0.112). There was a significant difference in the PALPA score (*Z* = −2.940, *p* < 0.05) but no significant difference (*Z* = 1.414, *p* = 0.157) in the PPTT.

### 3.3. Analysis of Error Patterns

The error patterns in the WAB-R object-naming task were analyzed. Results suggested that in the WAB-R English, during pre-treatment assessment, the participant produced a diverse set of errors: semantic errors (cup → glass), phonological related errors (rubber band → band), unrelated error (safety pin → pen), and no responses. There were also language transfer effects observed where a Malay word (matches → mancis/lighter) was produced in the English task. Similar error patterns were produced during post-treatment assessment.

During pre-treatment assessment in the WAB-R Malay, the participant made semantic error (getah ikat → rambut), phonological related error (pensil → pensin), neologism (pemadam → bodita), and circumlocutions (majority of the errors). Similar error patterns were observed in post-treatment assessment. However, it was noted that the participant made a number of cross-language effect errors when he produced English words (e.g., tukul → hammer, pemutar skru → screwdriver, sudu → spoon, berus gigi → toothbrush) instead of Malay words for object naming.

## 4. Discussion

The purpose of this pilot study was to examine the value of ACT as a treatment method to re-establish single-word writing in a bilingual patient with aphasia. Prior to treatment, GM showed minimal ability to write words in both English and Malay. During the ACT treatment, the patient demonstrated rapid learning for written spelling of the words targeted in the treatment. Following the results of his bilingual language profile measured on the LEAP-Q test and his preference for reading in English, the study targeted the phonemic-graphemic conversion route and English was selected as the language for the dysgraphia ACT treatment.

The patient not only improved in written objects naming (nouns), but also in written actions naming (verbs) with a comparable incremental curve and a significant change at the first baseline and in each multiple-baseline. A similar result was reported by [29] who found that single word writing abilities improve after ACT single-word writing treatment.

Similar to previous studies that used ACT [12,13], the ACT protocol developed in the current study has demonstrated the potential value of relatively simple clinical procedures to treat aphasic patients with writing difficulties. Interestingly, there was some evidence of a generalized treatment effect to the untrained Malay language after a treatment protocol in English, a language requiring the access to the phoneme-grapheme conversation, a process often reported as impaired in people with aphasia. It is important for speech-language therapists (SLTs) who manage bilingual aphasia patients to consider language-driven properties and to capitalize on bilingual competence to better support a long-term recovery. This is in line with what was suggested by [12] who stated that investigating a bilingual patient with aphasia required profiling a detailed history of language acquisition and use both pre- and post-morbidity. Information regarding exposure and use of L2, age of acquisition of L2, the language contexts in which L2 is used frequently, preferred language for certain activities, etc., is an essential component to facilitate objective assessment of a patient’s pre stroke L2 abilities and the system that is probably involved in processing this particular language (declarative/procedural memory).

From our data, it was observed that cross-language effects do occur particularly when GM is answering questions in Malay WAB-R. For this task, the patient tended to bring in words from English, into Malay. The information regarding L1-L2 grouping of languages for the patient is crucial to know, particularly for Malay individuals with aphasia living in a multilingual environment. ACT has served to strengthen specific graphemic representations and the ability to access them [17,18]. After the ACT treatment, GM was able to master the spelling of words of varying lengths, suggesting adequate function of the graphemic buffer. Assuming that graphemic representations were strengthened by the writing treatment, the associated semantic information must also be available in order for single-word writing to be used meaningfully in the other language.

In conclusion, single-word writing treatment may improve dysgraphia among adults with aphasia through the administration of a structured and systematic treatment in the language more dominant for that function and considering both specific factors at play in the language and the specific bilingual use of the language. The item-specific treatment of single-word spelling using ACT proposed in this study also considered specific pattern of use of words, which is the first step in designing a multiple-staged treatment plan. More data collection of this aspect of treatment should be carried out on different patients tracking their bilingual language profiles first, their specificity of the functional language use, and making use of the pervasive multilingual context of Malaysia to determine the best language for ACT protocols.

## Figures and Tables

**Table 1:** Results of Western Aphasia Battery-Revised (WAB-R) and Pyramid Palm Tree Test (PPTT) pre-and post-treatment.

	Pre-Treatment English (%)	Post-Treatment English (%)	Score Difference (%)	Pre-Treatment Malay (%)	Post-Treatment Malay (%)	Score Difference (%)
**WAB scores (possible)**						
**Spontaneous Speech**						
*Content (10)*	8 (80.0)	9 (90.0)	+1 (10.0)	6 (60.0)	9 (90.0)	+3 (30.0)
*Fluency (10)*	5 (50.0)	9 (90.0)	+4 (40.0)	3 (30.0)	8 (80.0)	+5 (50.0)
**Auditory Comprehension**						
*Y/N Questions (60)*	54 (90.0)	58 (96.7)	+4 (6.7)	51 (85.0)	54 (90.0)	+3 (5.0)
*Word Recognition (60)*	54 (90.0)	59 (98.3)	+5 (8.3)	50 (83.3)	60 (100.0)	+10 (16.7)
*Commands (80)*	72 (90.0)	76 (95.0)	+4 (5.0)	64 (80.0)	72 (90.0)	+8 (10.0)
**Repetition (100)**	62 (62.0)	80 (80.0)	+18 (18.0)	72 (72.0)	80 (80.0)	+8 (8.0)
**Naming**						
*Objects (60)*	37 (61.7)	53 (88.3)	+16 (26.6)	7 (11.7)	44 (73.3)	+34 (61.6)
*Verbal Fluency (20)*	3 (15.0)	9 (45.0)	+6 (30.0)	3 (15.0)	7 (35.0)	+4 (20.0)
*Sentence Completion (10)*	9 (90.0)	10 (100.0)	+1 (10.0)	5 (50.0)	9 (90.0)	+4 (40.0)
*Responsive Speech (10)*	10 (100.0)	10 (100.0)	0 (0.0)	9 (90.0)	10 (100.0)	+1 (10.0)
**Aphasia Quotient**	68.2	85.7	+17.5	53.7	82.6	+28.9
**Aphasia Type**	Conduction	Anomic	na	Broca’s	Anomic	na
**Severity**	Moderate	Mild		Moderate	Mild	
**Pyramid Palm Tree Test**						
*Pictures*	48 (92.3)	51 (98.1)	+3 (5.8)	na	na	na
*Written Words*	47 (90.4)	50 (96.2)	+3 (5.8)	na	na	na
**Treatment approach**	ACT	ACT	na	None	None	na

**Table 2:** Results of Psycholinguistics Assessment of Language Processing in Aphasia (PALPA).

Modality	Task	Test	Pre-Treatment (%)	Post-Treatment (%)	Score Difference (%)
Write	Writing words to dictation	PALPA 31	47 (58.8)	55 (68.8)	+8 (10.0)
Write	Spelling to dictation: Letter length	PALPA 39	14 (58.3)	20 (83.3)	+6 (25.0)
Write	Spelling to dictation: Imageability × Frequency	PALPA 40	27 (67.5)	38 (95.0)	+11 (27.5)
Write	Spelling to dictation: Grammatical class	PALPA 41	13 (65.0)	15 (75.0)	+2 (10.0)
Write	Spelling to dictation: Grammatical class × Imageability	PALPA 42	11 (55.0)	15 (75.0)	+4 (20.0)
Write	Spelling to dictation: Morphological endings	PALPA 43	18 (30.0)	40 (66.7)	+22 (36.7)
Write	Spelling to dictation: Regularity	PALPA 44	24 (60.0)	31 (77.5)	+7 (17.5)
Write	Spelling to dictation: Nonwords	PALPA 45	0 (0.0)	2 (8.3)	+2 (8.3)
Write	Spelling to dictation	PALPA 46	11 (55.0)	11 (55.0)	0 (0.0)
Write	Written picture naming	PALPA 53	27 (67.5)	36 (90.0)	+9 (22.5)
Read	Lexical decision	PALPA 25	54 (90.0)	57 (95.0)	+3 (5.0)
Read	Match written word to picture	PALPA 48	35 (87.5)	39 (97.5)	+4 (10.0)

## Appendix A

Words selected for treatment (trained words). All words included in each set covered a predicted set of properties to match difficulties across sets (incremental frequency and length) calculated on MRC Psycholinguistics database [30].

**Table A1:** List of trained words selected for the treatment of dysgraphia.

	Set 1		Set 2		Set 3	
	Nouns	Verbs	Nouns	Verbs	Nouns	Verbs
1.	Tissue	Wrap	Cabbage	Type	Fries	Bake
2.	Pizza	Squeeze	Globe	Paste	Squash	Grate
3.	Cricket	Tear	Gymnastic	Swim	Toaster	Spread
4.	Perfume	Tickle	Chocolate	Shave	Onion	Hug
5.	Dominos	Yawn	Dice	Search	Kettle	Pray
6.	Sandwich	Break	Purse	Wave	Fridge	Tire
7.	Pear	Bite	Archery	Pinch	Ladder	Spray
8.	Pineapple	Vacuum	Salad	Complete	Drawer	Arrange
9.	Kite	Count	Chips	Weigh	Carpet	Spill
10.	Luggage	Sweep	Hanger	Stroke	Torch	Smell
